# Predicting severe COVID-19 in elderly patients using routine laboratory indicators: Diagnostic accuracy of machine learning models

**DOI:** 10.1097/MD.0000000000049829

**Published:** 2026-07-17

**Authors:** Wenlan Lai, Yanbin Lin, Gaofeng Ou, Xuejun Qin, Ping Yang, Rongyan Chen

**Affiliations:** a Department of Clinical Laboratory, People’s Hospital Affiliated to Fujian University of Traditional Chinese Medicine, Fuzhou, Fujian, P. R. China; b Department of Respiratory Medicine, People’s Hospital Affiliated to Fujian University of Traditional Chinese Medicine, Fuzhou, Fujian, P. R. China; c The First Clinical Medical College, Fujian University of Traditional Chinese Medicine, Fuzhou, Fujian, P. R. China.

**Keywords:** COVID-19, elderly, laboratory indicators, machine learning, predictive model

## Abstract

To enable early warning of severe 2019 coronavirus disease in elderly patients, this study collected routine laboratory indicators and clinical parameters to construct and validate clinical models to predict the risk of severe disease. A total of 123 elderly 2019 coronavirus disease patients (68 non-severe and 55 severe) were retrospectively enrolled and randomly split into training and test sets. Predictive variables were selected via univariate analysis and stepwise logistic regression (LR) with variance inflation factor testing. Four models – LR, Random Forest, Support Vector Machine (SVM), and eXtreme Gradient Boosting Tree – were built. Model stability was assessed with 100 rounds of Bootstrap resampling. Performance was evaluated using receiver operating characteristic curves, calibration curves, and decision curve analysis in the test set. Five variables were selected: lymphocyte percentage, red blood cell count, total iron-binding capacity, unsaturated iron-binding capacity, and red cell distribution width. Multivariate LR identified lymphocyte percentage, red blood cell count, and total iron-binding capacity as protective factors, while unsaturated iron-binding capacity and red cell distribution width as risk factors. Bootstrap showed SVM and LR had superior stability. In the test set, SVM achieved the highest area under the curve (best discrimination); Random Forest showed best calibration; LR and eXtreme Gradient Boosting yielded slightly higher net benefits. Based on routine laboratory indicators, 4 prediction models were constructed and compared. These models can quantify severe risk using routine data early after admission, demonstrating strong clinical application potential.

## 1. Introduction

2019 Coronavirus disease (COVID-19) is a major global public health event caused by severe acute respiratory syndrome coronavirus 2 (SARS-CoV-2). Its main clinical manifestations include fever, cough, shortness of breath, and sore throat. Elderly patients are more likely to progress to severe and critical illness after infection, with higher severity and mortality rates.^[1]^

Regarding epidemic trends and public health threats, studies have shown that the case fatality rate among COVID-19 patients over 60 years old in China is significantly higher than in younger groups, reaching 14.8% in those over 80 years old,^[2]^ posing a severe public health challenge. In terms of clinical characteristics, elderly patients often have multiple comorbidities. Existing research reveals that advanced age, comorbidities such as hypertension, diabetes, and cardiovascular diseases, as well as immunosenescence, are multidimensional risk factors for disease progression to severe illness in elderly patients.^[3,4]^ Pathologically, immune dysregulation and excessive inflammatory response are core mechanisms driving disease deterioration. Elderly patients are more prone to laboratory abnormalities such as decreased lymphocyte count, reduced hemoglobin,^[5]^ elevated inflammatory markers (such as neutrophil to lymphocyte ratio and interleukin-6),^[6,7]^ coagulation dysfunction (elevated d-dimer),^[8]^ and hypoalbuminemia.^[9]^ In summary, complete blood count and routine biochemical indicators possess strong clinical universality, convenient testing, and rapid result turnaround, occupying an important position in early warning of severe COVID-19 risk in elderly patients. However, individual indicators often have limited predictive power. Therefore, a predictive model combining multiple clinical and laboratory parameters could serve as an index test for early identification of elderly COVID-19 patients at high risk of progressing to severe disease. Its intended clinical use is to support risk stratification and facilitate timely intervention.

In this study, we constructed COVID-19 severe disease risk prediction models through machine learning, aiming to utilize joint modeling to mine hidden warning signals from complex and massive clinical laboratory indicators and clinical parameters in the big data era, providing new potential and possibilities for solving clinical dilemmas.

## 2. Materials and methods

### 2.1. Study subjects

This study employed a retrospective cohort design. A consecutive series of 123 elderly patients with confirmed COVID-19, admitted to our hospital from March 2024 to July 2025, were enrolled as study participants, and 47 laboratory indicators at admission were analyzed. Demographic characteristics (age and gender) and 10 comorbidities (diabetes mellitus, chronic respiratory disease, chronic heart failure, coronary heart disease, chronic liver disease, chronic kidney disease, cancer, hypertension, cerebral infarction, and immune-mediated disease) were also collected.

All enrolled patients met the clinical diagnostic criteria in the *Diagnosis and Treatment Protocol for Novel Coronavirus Infection (Trial Version 10*). Inclusion criteria: clinical manifestations of novel coronavirus infection; positive novel coronavirus antigen/nucleic acid test at admission; and age over 60 years, regardless of gender. Exclusion criteria: history of hematological diseases or red blood cell transfusion within 3 months and insufficient diagnostic evidence or incomplete clinical data. Patients’ clinical classification at discharge was based on the *Diagnosis and Treatment Protocol for Novel Coronavirus Infection (Trial Version 10*), divided into mild, moderate, severe, and critical types.^[10]^ This study classified mild and moderate types as the non-severe group, and severe and critical types as the severe group. This study was approved by the Ethics Committee of People’s Hospital Affiliated to Fujian University of Traditional Chinese Medicine (2023-046-01), and informed consent was obtained from all participants.

### 2.2. Data collection

Twenty-four indicators were obtained from complete blood count results detected by Mindray Hematology Analyzer (BC7500): white blood cell count, absolute neutrophil count, absolute lymphocyte count, absolute monocyte count, absolute eosinophil count, absolute basophil count, neutrophil percentage, lymphocyte percentage (LY%), monocyte percentage, eosinophil percentage, basophil percentage, red blood cell count (RBC), hemoglobin, hematocrit, mean corpuscular volume, mean corpuscular hemoglobin, mean corpuscular hemoglobin concentration, red cell distribution width (RDW), platelet count, mean platelet volume, plateletcrit, platelet distribution width, large platelet ratio, and high-sensitivity C-reactive protein.

Twenty-three indicators were obtained from routine biochemical results detected by the biochemical immunoassay integrated system (Abbott C16000): total protein, albumin, globulin, alanine aminotransferase, aspartate aminotransferase, total bilirubin, serum direct bilirubin, serum indirect bilirubin, creatinine, uric acid, urea, total cholesterol, high-density lipoprotein cholesterol, low-density lipoprotein cholesterol, immunoglobulin A, immunoglobulin G, immunoglobulin M, complement C3 (C3), complement C4 (C4), total iron-binding capacity (TIBC), unsaturated iron-binding capacity (UIBC), serum iron, and ferritin.

All measurements were performed according to the standard operating procedures. To ensure test reproducibility, routine internal quality control samples were analyzed in each run, and all quality control results were within acceptable ranges as specified by the manufacturer. The reference ranges for each indicator were established based on the laboratory’s validated protocols and the manufacturers’ recommendations, allowing the experimental results to be reproducible and verifiable by other researchers.All laboratory technicians performing the index tests were blinded to the patients’ clinical information, including their disease severity status, other test results, and final diagnosis.

### 2.3. Research design

Data were independently entered by 2 researchers and cross-checked. This study had complete data with no missing values. The dataset was randomly divided into training and test sets at a 7:3 ratio using random sampling, ensuring balanced distribution of “severe” and “non-severe” cases in both sets. Feature selection was conducted in 2 steps: First, univariate analysis (*P* < .05) was performed on candidate variables in the training set; second, stepwise logistic regression (LR; with minimum Akaike information criterion as criterion) was performed on preliminarily screened variables, and variance inflation factor (VIF > 10) was used to diagnose and exclude variables with multicollinearity. The final determined variables were used for subsequent modeling.

Four machine learning algorithms were used to construct predictive models: Multivariate LR: modeling based on screened variables to assess their independent contribution. Random Forest (RF): optimizing main hyperparameters (such as ntree, mtry) through grid search and 10-fold cross-validation, and assessing variable importance. Support Vector Machine (SVM): Using Radial Basis Function kernel, optimizing penalty parameter C and kernel parameter σ through cross-validation. eXtreme Gradient Boosting (XGBoost) Tree: optimizing key hyperparameters such as learning rate and maximum depth through cross-validation, and quantifying feature importance. Bootstrap resampling was used for internal validation during modeling.

Model performance was comprehensively evaluated in the test set from 3 dimensions: discrimination, calibration, and clinical utility. Specific indicators included: area under the receiver operating characteristic curve (AUC), accuracy, sensitivity, specificity, F1 score, as well as calibration curves and decision curve analysis.

### 2.4. Statistical analysis

Data analysis and visualization were performed in R language (v4.3.3) environment, using main packages including dplyr, caret, glmnet, randomForest, e1071, xgboost, pROC, and ggplot2. Categorical variables were compared using Chi-square test or Fisher exact test, and continuous variables between groups were compared using Mann–Whitney *U* test. For data with detection of limits (LOD), values below LOD were uniformly assigned the detection limit value before nonparametric testing. Continuous variables following normal distribution were expressed as $\bar{x}\pm s$, while those not following normal distribution were expressed as M (Q1, Q3). *P* < .05 was considered statistically significant. Wald test was used in univariate regression analysis for each variable, and variables with *P* < .05 were included in stepwise regression for further screening. Wald test was used in multivariate LR analysis, and variables with *P* < .05 were considered independent predictors. AUC and its 95% CI were estimated through Bootstrap method to improve result robustness and reflect interval uncertainty.

## 3. Results

### 3.1. Patient basic characteristics and laboratory parameter

Among 123 confirmed elderly COVID-19 patients, clinical classification at discharge was: 68 cases in the non-severe group and 55 cases in the severe group. No significant differences existed between the 2 groups in gender ratio and age. Significant differences (*P* < .05) were observed in the prevalence of chronic heart failure (Table 1) and 23 laboratory indicators (Table 2) showed significant differences, including white blood cell count, absolute neutrophil count, neutrophil percentage, LY%, monocyte percentage, eosinophil percentage, basophil percentage, RBC, hemoglobin, hematocrit, RDW, high-sensitivity C-reactive protein, albumin, alanine aminotransferase, aspartate aminotransferase, C3, serum iron, high-density lipoprotein cholesterol, immunoglobulin G, TIBC, total protein, UIBC, and urea. The other 9 comorbidities and 24 indicators showed no statistical differences between the 2 groups (*P* > .05; Table S1, Supplemental Digital Content 1).

**Table 1:** Demographic and comorbidities of patient.

Parameters	Non-severe group (n = 68)	Severe group (n = 55)	*P*
Age (yrs)	75 (68–82)	76 (70–84)	.465*
Gender			
Male	49 (72.1%)	38 (69.1%)	.719†
Female	19 (27.9%)	17 (30.9%)	
Comorbidities, n(%)			
Diabetes mellitus	22 (32.4%)	18 (32.7%)	.965†
Chronic respiratory disease	20 (29.4%)	22 (40%)	.218†
Chronic heart failure	8 (11.8%)	14 (25.5%)	.049†
Cardiovascular disease	17 (25%)	12 (21.8%)	.679†
Chronic liver disease	4 (5.9%)	3 (5.5%)	.919†
Chronic kidney disease	10 (14.7%)	13 (23.6%)	.207†
Cancer (any)	16 (23.5%)	12 (21.8%)	.822†
Hypertension	38 (55.9%)	33 (60%)	.646†
Cerebral infarction	20 (29.4%)	20 (36.4%)	.413†
Immune-mediated disease	6 (8.8%)	3 (5.5%)	.476†

* Mann–Whitney *U* test.

† Chi-square Test.

**Table 2:** Laboratory indicators of patient.

Parameters	Non-severe group (n = 68)	Severe group (n = 55)	*P*
WBC (*×*10^3^/μL)	6.75 (5.10–8.10)	9.30 (6.60–11.60)	.001†
NE (*×*10^3^/μL)	4.60 (3.20–6.13)	7.60 (4.65–9.85)	*<*.001†
NE%	71.45 (64.08–78.00)	79.90 (71.10–88.25)	*<*.001†
LY%	16.30 (11.15–24.80)	11.60 (6.60–18.95)	.001†
MO%	9.25 (7.48–10.93)	7.10 (4.40–9.95)	.002†
EO%	1.20 (0.30–1.95)	0.40 (0.10–1.10)	.008†
BA%	0.30 (0.20–0.50)	0.20 (0.10–0.40)	.005†
RBC (*×*10^6^/μL)*	3.87 *± *0.73	3.21 *± *0.74	*<*.001†
HB (g/L)*	115.99 *± *20.39	97.98 *± *21.66	*<*.001†
HCT*	0.34 *± *0.06	0.29 *± *0.06	*<*.001†
RDW (%)	13.25 (12.48–14.30)	14.30 (13.00–16.30)	.002†
hs-CRP (mg/L)	17.25 (9.76–44.15)	29.60 (18.00–88.16)	.004†
ALB (g/L)*	35.16 *± *5.52	30.60 *± *4.00	*<*.001†
ALT (U/L)	16.00 (11.00–25.00)	23.00 (15.00–40.50)	.003†
AST (U/L)	21.50 (17.00–30.00)	27.00 (20.00–47.00)	.007†
C3 (g/L)*	1.32 *± *0.33	1.17 *± *0.37	.016†
FER (ng/mL)	376.09 (253.68–544.56)	810.91 (360.95–2238.89)	*<*.001†
HDL-C (mmol/L)	1.04 (0.81–1.31)	0.86 (0.60–1.02)	.002†
IgG (g/L)	13.01 (9.97–14.72)	10.49 (8.48–13.00)	.008†
TIBC (μmol/L)	45.85 (41.40–54.83)	38.10 (28.95–43.25)	*<*.001†
TP (g/L)	62.75 (57.98–67.73)	56.50 (52.40–61.90)	*<*.001†
UIBC (μmol/L)	34.50 (27.43–45.20)	25.30 (18.50–34.10)	*<*.001†
Urea (mmol/L)	5.63 (4.13–7.74)	7.68 (5.38–11.25)	.002†

Laboratory indicators with *P* < .05.

ALB = albumin, ALT = alanine aminotransferase, AST = aspartate aminotransferase, BA% = basophil percentage, C3 = complement C3, EO% = eosinophil percentage, FER = ferritin, HB = hemoglobin, HCT = hematocrit, HDL-C = high-density lipoprotein cholesterol, hs-CRP = high-sensitivity C-reactive protein, IgG = immunoglobulin G, LY% = lymphocyte percentage, MO% = monocyte percentage, NE = absolute neutrophil count, NE% = neutrophil percentage, RBC = red blood cell count, RDW = red cell distribution width, TIBC = total iron-binding capacity, TP = total protein, UIBC = unsaturated iron-binding capacity, WBC = white blood cell count.

* Mean±SD.

† Mann–Whitney *U* Test.

### 3.2. Variable selection results

Data were divided into training set (87 cases) and test set (36 cases) at a 7:3 ratio using random sampling, with balanced distribution of “non-severe” and “severe” cases in both sets. Univariate regression analysis was performed on the training set, screening out 19 variables with *P* < .05 (Table 3), which participated in stepwise LR. The other 40 variables (gender, age, 10 comorbidities, and 28 laboratory indicators) were excluded (Table S2, Supplemental Digital Content 2).

**Table 3:** Univariate regression analysis.

Risk factor	*B*	S.E.	Wald	*P*	OR (95% CI)
RBC	−1.382	0.386	12.829	<.001	0.251 (0.118–0.535)
HB	−0.051	0.014	13.766	<.001	0.951 (0.926–0.976)
HCT	−16.998	4.643	13.404	<.001	0 (0–0)
TIBC	−0.105	0.028	14.171	<.001	0.900 (0.852–0.951)
ALB	−0.181	0.054	11.153	.001	0.835 (0.751–0.928)
TP	−0.107	0.033	10.287	.001	0.898 (0.841–0.959)
NE%	0.067	0.022	9.256	.002	1.069 (1.024–1.116)
LY%	−0.088	0.030	8.796	.003	0.915 (0.863–0.970)
UIBC	−0.058	0.019	8.979	.003	0.944 (0.909–0.980)
NE	0.165	0.063	6.887	.009	1.180 (1.043–1.335)
WBC	0.137	0.057	5.799	.016	1.147 (1.026–1.282)
hs-CRP	0.012	0.005	5.801	.016	1.012 (1.002–1.022)
HDL-C	−1.505	0.641	5.521	.019	0.222 (0.063–0.779)
RDW	0.257	0.111	5.339	.021	1.293 (1.040–1.609)
TC	−0.409	0.179	5.232	.022	0.664 (0.468–0.943)
C3	−1.436	0.656	4.796	.029	0.238 (0.066–0.860)
Urea	0.114	0.053	4.672	.031	1.121 (1.011–1.243)
IgG	−0.122	0.057	4.594	.032	0.885 (0.791–0.990)
FER	0	0	4.501	.034	1.000 (1.000–1.001)

Variables with *P* < .05 included in stepwise regression.

ALB = albumin, C3 = complement C3, FER = ferritin, HB = hemoglobin, HCT = hematocrit, HDL-C = high-density lipoprotein cholesterol, hs-CRP = high-sensitivity C-reactive protein, IgG = immunoglobulin G, LY% = lymphocyte percentage, NE = absolute neutrophil count, NE% = neutrophil percentage, RBC = red blood cell count, RDW = red cell distribution width, TC = total cholesterol, TIBC = total iron-binding capacity, TP = total protein, UIBC = unsaturated iron-binding capacity, WBC = white blood cell count.

Through stepwise LR with VIF testing, the variables included in the model with minimum Akaike information criterion were determined as LY%, RBC, TIBC, UIBC, and RDW, with VIF values ranging between 1.017 and 1.053 (all < 10), and were included in subsequent modeling (Table 4).

**Table 4:** Multivariate logistic regression analysis model.

Risk factor	*B*	S.E.	Wald	*P*	OR (95% CI)
LY%*	−0.086	0.036	5.578	.018	0.918 (0.850–0.982)
RBC*	−0.963	0.463	4.316	.038	0.382 (0.144–0.914)
TIBC*	−0.152	0.058	6.894	.009	0.859 (0.759–0.955)
UIBC	0.069	0.044	2.386	.122	1.071 (0.984–1.175)
RDW	0.188	0.135	1.941	.164	1.207 (0.942–1.625)
(Intercept)	6.284	3.193	3.873	.049	535.92 (1.251–423,470.568)

LY% = lymphocyte percentage, RBC = red blood cell count, RDW = red cell distribution width, TIBC = total iron-binding capacity, UIBC = unsaturated iron-binding capacity.

* Independent predictors.

### 3.3. Model construction

Four methods were used to construct models: LR, RF, SVM, and XGBoost. The core parameters after optimization for each model were as follows: RF (ntree = 500, mtry = 5); SVM (kernel σ=0.01, penalty coefficient C = 1); and XGBoost (nrounds = 100, max_depth = 7, eta = 0.05, subsample = 0.9). Other parameters used default settings.

Internal validation was performed using the Bootstrap method during modeling. The training set was resampled 100 times, models were rebuilt each time, and the median AUC and 95% CI (2.5% ~ 97.5%) were calculated on corresponding Bootstrap test samples. All 4 models showed good stability. Among them, SVM and LR had higher median AUC values, with the 97.5th percentile still >0.7, showing superior and more stable performance compared to RF and XGBoost (Table 5).

**Table 5:** Performance comparison of various models.

Model	AUC	Accuracy	Sensitivity	Specificity	F1 score	H-L test*	MAE	Net benefit†	Model stability‡
LR	0.794	0.750	0.684	0.824	0.734	0.024	0.330	0.366	0.787 (0.747–0.820)
RF	0.763	0.667	0.750	0.600	0.667	0.052	0.056	0.338	0.753 (0.669–0.832)
SVM	0.813	0.778	0.750	0.800	0.750	0.694	0.355	0.358	0.797 (0.758–0.831)
XGBoost	0.806	0.694	0.813	0.600	0.703	0.346	0.340	0.366	0.764 (0.694–0.843)

AUC = area under the curve, LR = logistic regression, MAE = mean absolute error, RF = Random Forest, SVM = Support Vector Machine, XGBoost = eXtreme Gradient Boosting.

* Hosmer–Lemeshow test *P*-value.

† Net benefit at the threshold range of 0.15.

‡ Median AUC and 95% confidence interval after Bootstrap validation.

The prediction formula for LR was:


$$\begin{array}{c} \begin{array}{cc} & y=-0.086\times \left(LY\%\right)-0.963\times \left(RBC\right)\\ & -0.152\times \left(TIBC\right)+0.069\times \left(UIBC\right)\\ & +0.188\times \left(RDW\right)+6.284. \end{array} \end{array}$$

**Figure 1.**
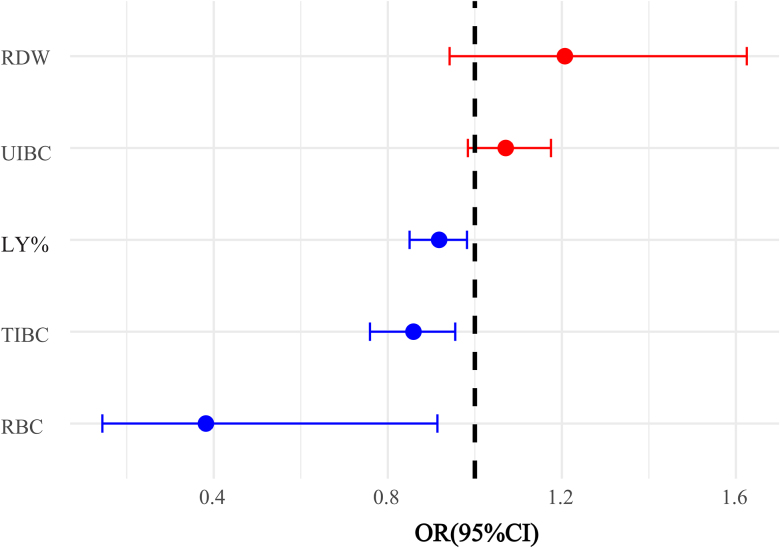
Forest plot of multivariate logistic regression. LY% = lymphocyte percentage, RBC = red blood cell count, RDW = red cell distribution width, TIBC = total iron-binding capacity, UIBC = unsaturated iron-binding capacity.

Among them, LY% (OR = 0.918, *P* = .018), RBC (OR = 0.382, *P* = .038), and TIBC (OR = 0.859, *P* = .009) were independent predictors. UIBC (OR = 1.071, *P* = .122) and RDW (OR = 1.207, *P* = .164) did not reach statistical significance in the LR model. LY%, RBC, and TIBC were protective factors, while UIBC and RDW were risk factors (Table 4, Fig. 1).

**Figure 2.**
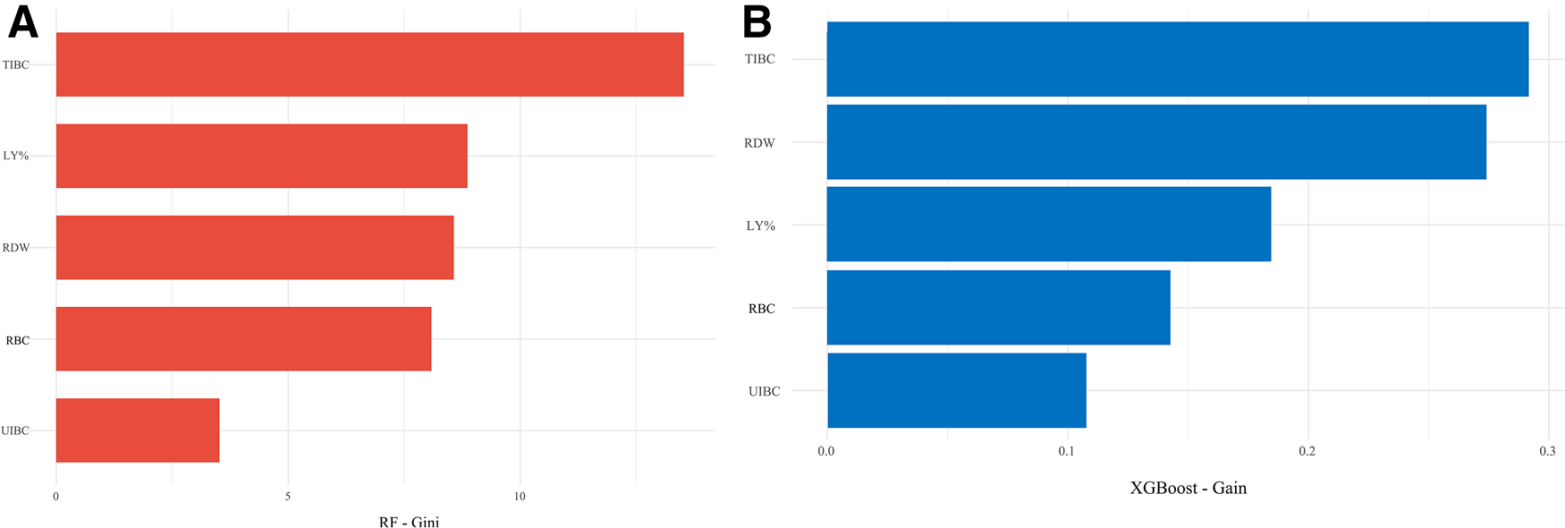
Importance ranking of the 5 variables: The left panel (A) shows the Gini importance derived from the Random Forest model, representing the mean decrease in node impurity. The right panel (B) displays the importance scores (measured by Gain) from the XGBoost model, indicating the average improvement in accuracy when a feature is used for splitting. LY% = lymphocyte percentage, RBC = red blood cell count, RDW = red cell distribution width, TIBC = total iron-binding capacity, UIBC = unsaturated iron-binding capacity, XGBoost = eXtreme Gradient Boosting.

The variable importance rankings from RF and XGBoost models provided additional information. In the RF model, the Gini scores from high to low were TIBC (13.52), LY% (8.86), RDW (8.57), RBC (8.09), and UIBC (3.52). In the XGBoost model, the importance scores from high to low were TIBC (0.292), RDW (0.274), LY% (0.185), RBC (0.143), and UIBC (0.107; Fig. 2).

### 3.4. Model evaluation

#### 3.4.1. Discrimination evaluation of models

**Figure 3.**
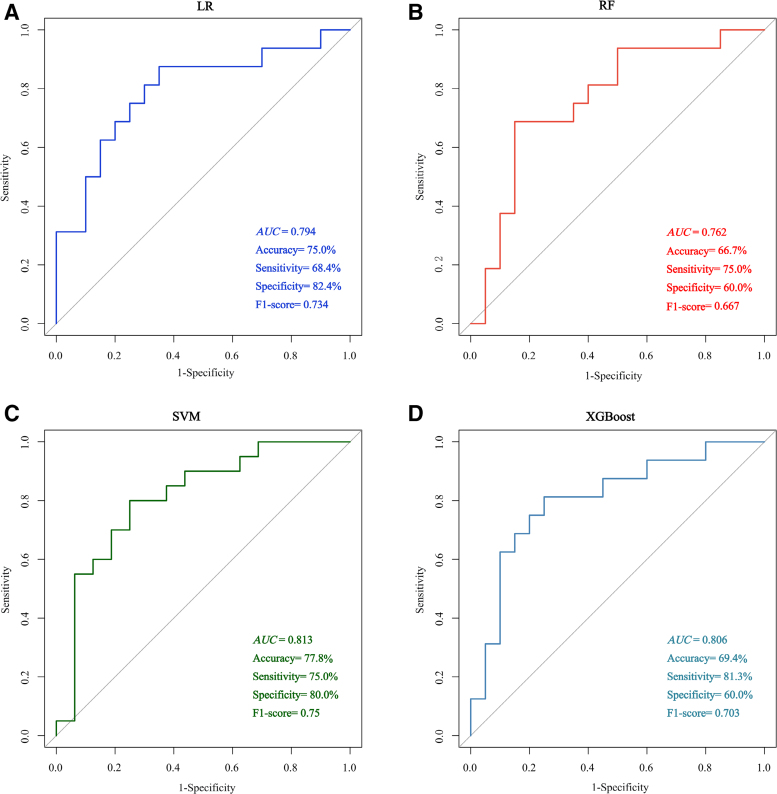
Comparison of ROC curves for 4 models in test set (A–D). AUC = area under the curve, LR = logistic regression, RF = Random Forest, ROC = receiver operating characteristic, SVM = Support Vector Machine, XGBoost = eXtreme Gradient Boosting.

In the test set, the AUC values for the LR, RF, SVM, and XGBoost models were 0.794, 0.762, 0.813, and 0.806, respectively. Overall, SVM showed the best discrimination (Fig. 3, Table 5).

#### 3.4.2. Calibration evaluation of models

**Figure 4.**
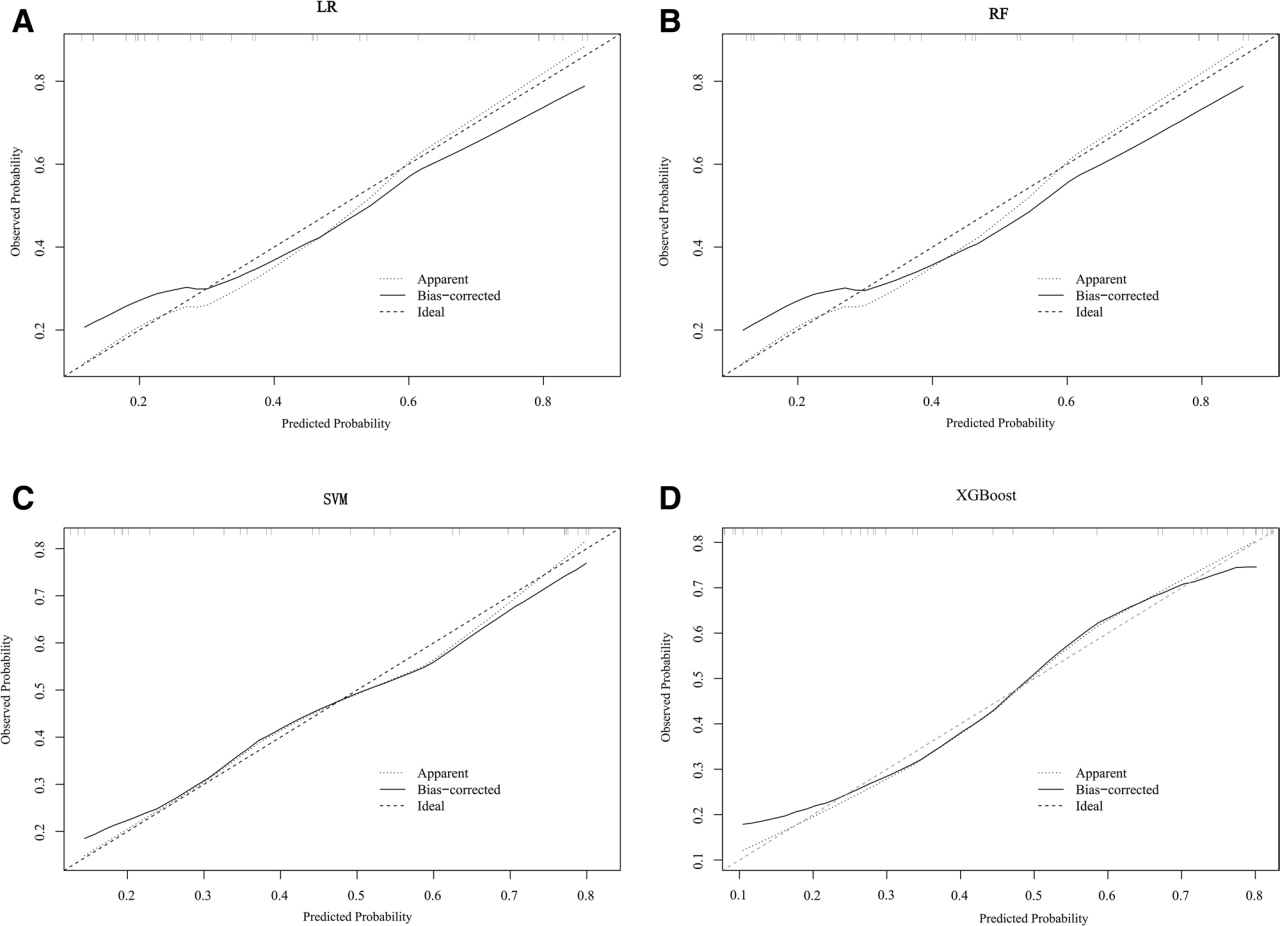
Calibration curves for 4 models in test (A–D). The ideal diagonal represents perfect calibration curve. Apparent represents calibration curve fitted to original data. Bias-corrected represents calibration curve after resampling correction for overfitting bias. LR = logistic regression, RF = Random Forest, SVM = Support Vector Machine, XGBoost = eXtreme Gradient Boosting.

Calibration curves were used to assess the consistency between predicted probabilities and actual observed risks. The calibration curves for all 4 models in the test set were close to the diagonal line, indicating good consistency between predicted probabilities and actual risks. The Hosmer–Lemeshow test for LR suggested overfitting (*P* < .05). Among the 4 models, RF had the smallest mean absolute error, indicating the best calibration (Fig. 4, Table 5).

#### 3.4.3. Clinical utility evaluation of models

**Figure 5.**
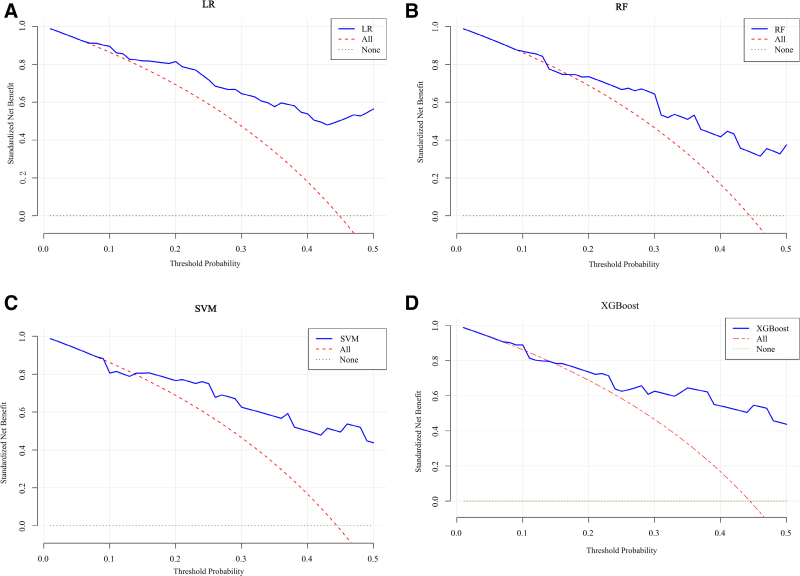
Decision curve analysis for 4 models in test (A–D). The blue solid line represents the prediction model, the red dashed line “All” represents the “treat all” strategy, and the green dashed line “None” represents the “treat none” strategy. In this model, “intervention” for patients clinically refers to close observation or even intensive care; “treat all” means wasting medical resources, while “treat none” means missing severe patients who need attention. LR = logistic regression, RF = Random Forest, SVM = Support Vector Machine, XGBoost = eXtreme Gradient Boosting.

Decision curve analysis was used to assess clinical net benefit of models at different threshold probabilities in the test set. All 4 models showed positive net benefit within the threshold probability range of 0.10 to 0.50, higher than the 2 extreme strategies of “treat all” and “treat none,” indicating good clinical utility. The optimal threshold range for each model was 0.10 to 0.30. At threshold range of 0.15, LR and XGBoost showed slightly higher net benefits than SVM and RF (Fig. 5, Table 5).

## 4. Discussion

### 4.1. Model construction and evaluation

This study constructed and validated severe disease risk warning models for elderly COVID-19 patients based on 47 laboratory indicators and clinical parameters (gender, age, and 10 comorbidities). No significant differences existed between the 2 groups in gender and age. Through analysis, the prevalence of chronic heart failure and 23 laboratory indicators showed significant differences between non-severe and severe groups. 19 variables were screened out through univariate analysis, and finally 5 predictive variables were determined through stepwise LR: LY%, RBC, TIBC, UIBC, and RDW. Among them, LY%, RBC, and TIBC were protective factors, while UIBC and RDW were risk factors. LR results showed that LY%, RBC, and TIBC were independent predictors; although UIBC and RDW did not reach statistical significance, they could still serve as auxiliary warning indicators. The variable importance rankings from RF and XGBoost models further suggested that TIBC ranked first. This study used LR, SVM, RF, and XGBoost to construct classification models and performed internal validation based on Bootstrap showing good stability for all models. Test set evaluation showed that each model had complementary advantages: SVM showed the best discrimination, RF showed the best calibration, while LR and XGBoost showed slightly higher net benefits in clinical utility.

### 4.2. Application scenarios of models

The performance characteristics of the above models provide basis for algorithm selection in clinical scenarios. Overall, LR can be the preferred tool for clinical decision support due to its intuitive model coefficients, strong interpretability, optimal calibration performance, and ease of clinical calculation. If clinical scenarios focus on high sensitivity, such as ICU resource prediction or rapid triage in fever clinics, XGBoost (sensitivity 81.3%) or SVM (accuracy 77.8%) can be prioritized, as both have advantages in identifying high-risk patients.^[11]^ In addition, although RF showed slightly inferior overall performance compared to other models with small sample data in this study, it has good robustness for high-dimensional data and missing values, making it more suitable for application in multicenter, multi-indicator big data.^[12]^ In practical applications, flexible selection or model fusion can be made according to specific clinical needs, decision preferences, and data conditions.

### 4.3. Significance of predictive variables

Lymphocytopenia is one of the core indicators for predicting severe disease progression. SARS-CoV-2 infection can induce a state of excessive inflammatory response coexisting with immune deficiency.^[13]^ The mechanism may involve direct infection of lymphocytes by the virus leading to apoptosis, as well as inhibition of bone marrow hematopoietic function by inflammatory storms, resulting in reduced lymphocyte production.^[14]^ Studies have shown that the degree of lymphocyte reduction is significantly correlated with the severity and prognosis of COVID-19. In elderly patients, due to age-related thymic degeneration and decreased T cell output, as well as preexisting chronic low-grade inflammation, the baseline lymphocyte count is already low, making it more vulnerable to the impact of SARS-CoV-2 infection, leading to a sharp decline in lymphocyte count and subsequent immune collapse.^[15]^ Therefore, early dynamic monitoring of LY% is crucial for identifying elderly patients at risk of progressing to severe disease.

Changes in the erythrocyte system provide another important warning perspective. Decreased RBC and elevated RDW are positively correlated with disease severity. The mechanism stems from systemic inflammation. On one hand, inflammatory factors inhibit bone marrow erythropoiesis and interfere with iron metabolism, leading to anemia and increased erythrocyte volume heterogeneity.^[16]^ On the other hand, oxidative stress accelerates erythrocyte destruction, leading to elevated RDW.^[17,18]^ In addition, erythrocytes themselves actively participate in immune pathological processes, and functional changes in surface receptors (such as TLR9 and CR1) can exacerbate the disorder of inflammatory clearance and erythrocyte self-destruction.^[19]^ RDW not only reflects anemia status but is also a comprehensive indicator of inflammatory load and oxidative damage. Due to commonly accompanied decreased baseline immune function and chronic low-grade inflammatory state, the erythrocyte metabolism and immune response of elderly patients are more susceptible to viral invasion and inflammatory storm impact. Their erythrocyte metabolism and immune response are more easily affected by viral invasion and inflammatory storm. Their fragile immune homeostasis after infection is easily disrupted, with strong inflammatory reactions further exacerbating the dual blow to erythropoiesis and survival.Therefore, dynamic monitoring of RBC and RDW levels in elderly COVID-19 patients not only helps understand their severe disease progression from an immune-inflammatory perspective but also provides important laboratory warning information for clinical identification of high-risk patients and timely intervention to improve tissue oxygenation and prognosis.

Iron metabolism disorders reveal severe disease risk from a deeper metabolic perspective. Iron is a key element for oxygen transport, energy metabolism, and immune cell function. Studies have confirmed a close association between iron metabolism-related indicators and the severity of COVID-19, highlighting the clinical value of hyperferritinemia as a core predictor of disease progression to severe illness.^[20,21]^ COVID-19 patients often show decreased TIBC and iron homeostasis imbalance, which is directly related to severe inflammatory status. Hepatocyte synthesis of transferrin is inhibited, leading to decreased TIBC. More critically, virus-induced cytokine storms may exacerbate tissue damage through triggering “ferroptosis,” a novel cell death pathway,^[22]^ involving destruction of the GPX4 antioxidant system and ferritinophagy leading to intracellular iron overload.^[23]^ Notably, variable importance analysis based on RF and XGBoost in this study both showed TIBC with the highest importance score, suggesting its key role in iron metabolism and inflammatory regulation may have important warning value for COVID-19 severe disease progression. Elderly patients, due to inherent weakened iron metabolism regulation ability and chronic inflammation foundation, are more prone to iron redistribution abnormalities and ferroptosis activation after infection, thereby inducing higher organ damage risk and mortality.

In summary, lymphocyte, erythrocyte and iron metabolism indicators collectively construct an early warning system for assessing severe disease risk in COVID-19 patients, especially elderly patients, from 3 interconnected dimensions of immunity, inflammation, and metabolism. In clinical practice, joint and dynamic monitoring of these indicators in elderly COVID-19 patients not only helps identify high-risk individuals earlier and more comprehensively but also provides key laboratory basis and ideas for exploring targeted intervention strategies such as immune regulation, antioxidant therapy, and ferroptosis inhibition, holding important clinical significance for improving patient prognosis.

### 4.4. Strengths and limitations

The strengths of this study lie in: first, all indicators of the constructed models are derived from routine clinical laboratory tests, with easy data access, low cost, and rapid reporting, possessing good clinical applicability and promotion potential. Second, the study focuses on elderly high-risk populations, helping achieve risk stratification and targeted intervention. Third, the model structure is concise and intuitive, facilitating rapid application in clinical practice. However, this study still has certain limitations: as a single-center retrospective study, the sample size is relatively limited, and external validation is lacking, results may be affected by selection bias. Some hematological indicators in elderly populations are easily interfered by comorbidities, nutritional status, and medication use, which may affect model stability and generalization ability.

In conclusion, this study constructs and validates 4 machine learning models based on routine laboratory indicators for predicting severe disease risk in elderly COVID-19 patients. In clinical application, this model can provide doctors with an objective, quantitative risk warning tool, helping to accurately identify high-risk elderly patients in the early stage of disease, thereby timely initiating intensive monitoring and intervention.

## Acknowledgments

The authors thank Jieying Zhuang for her valuable contributions to data entry and laboratory operations. We also acknowledge the use of R language and related open-source packages for data analysis and visualization. This study was supported by the Project for Medical Technology Discipline of Fujian University of Traditional Chinese Medicine (Grant No. X202301 and XJJGY25107).

## Author contributions

**Conceptualization:** Wenlan Lai.

**Data curation:** Gaofeng Ou, Xuejun Qin, Ping Yang.

**Formal analysis:** Yanbin Lin, Rongyan Chen.

**Funding acquisition:** Rongyan Chen.




