# Supplementary material for: Predicting severe COVID-19 in elderly patients using routine laboratory indicators: Diagnostic accuracy of machine learning models
Source: Medicine (Baltimore). 2026 Jul 17;105(29):e49829. doi: 10.1097/MD.0000000000049829 (PMC13384693; doi:10.1097/MD.0000000000049829)
Supplement: Supplementary file 1 [file medi-105-e49829-s001.docx]

### ****Supplementary**** Table 1 The other 24 laboratory Indicators of Patient

| **Parameters** | **Non-severe group(n=68)** | **Severe group(n=55)** | **P** |
| --- | --- | --- | --- |
| LY (*×*10^3^ /*μ*L) | 1.10(0.80~1.50) | 0.90(0.60~1.50) | 0.199 *b* |
| MO (*×*10^3^ /*μ*L) | 0.60(0.40~0.70) | 0.60(0.40~0.90) | 0.499 *b* |
| EO (*×*10^3^ /*μ*L) | 0.10(0~0.20) | 0(0~0.10) | 0.092 *b* |
| BA (*×*10^3^ /*μ*L) | 0(0~0) | 0(0~0) | 0.442 *b* |
| MCV (fl) | 89.05(86.75~92.85) | 91.30(87.35~95.05) | 0.080 *b* |
| MCH (pg) | 30.30(29.25~31.33) | 30.90(29.65~31.90) | 0.136 *b* |
| MCHC (g/L) *a* | 337.78±11.18 | 338.04±12.28 | 0.904 *b* |
| PLT (*×*10^3^ /*μ*L) | 210.50(147.00~263.75) | 216.00(133.50~301.00) | 0.851 *b* |
| MPV (fl) | 9.80(9.18~10.50) | 9.70(9.20~10.40) | 0.873 *b* |
| PCT (%) | 0.205(0.16~0.253) | 0.21(0.14~0.29) | 0.996 *b* |
| PDW (%) | 12.20(10.18~15.93) | 10.60(9.45~16.15) | 0.448 *b* |
| P-LCR (%) *a* | 23.63±8.51 | 22.46±10.83 | 0.501 *b* |
| C4 (g/L) | 0.34(0.27~0.39) | 0.30(0.24~0.41) | 0.536 *b* |
| Crea(*μ*mol/L) | 82.50(68.75~106.75) | 71.00(56.00~116.50) | 0.130 *b* |
| DBIL(*μ*mol/L) | 2.35(1.70~3.13) | 2.70(1.90~3.75) | 0.202 *b* |
| SI(*μ*mol/L) | 10.05(6.80~16.90) | 8.90(6.30~14.45) | 0.391 *b* |
| GLO(g/L) | 28.40(24.40~30.43) | 26.60(22.70~29.05) | 0.131 *b* |
| IBIL(*μ*mol/L) | 3.95(2.10~6.28) | 3.30(1.90~6.50) | 0.357 *b* |
| IgA(g/L) | 2.58(1.69~3.38) | 2.00(1.39~3.04) | 0.223 *b* |
| IgM(g/L) | 0.94(0.64~1.31) | 0.90(0.62~1.28) | 0.923 *b* |
| LDL-C(mmol/L) | 2.23(1.51~2.87) | 2.06(1.51~2.62) | 0.537 *b* |
| TB(*μ*mol/L) | 6.10(3.90~8.78) | 6.10(4.05~9.95) | 0.915 *b* |
| TC (mmol/L) | 3.70(2.80~4.87) | 3.35(2.70~4.28) | 0.111 *b* |
| UA(*μ*mol/L) | 286.00(205.25~394.25) | 220.00(181.00~347.50) | 0.056 *b* |

Laboratory indicators with P>0.05. aMean±SD. bMann Whitney U Test. LY = absolute lymphocyte count, MO = absolute monocyte count, EO = absolute eosinophil count, BA = absolute basophil count, MCV = mean corpuscular volume, MCH = mean corpuscular hemoglobin, MCHC = mean corpuscular hemoglobin concentration, PLT = platelet count, MPV = mean platelet volume, PCT = plateletcrit, PDW = platelet distribution width, P-LCR = large platelet ratio, C4 = complement C4, DBIL = serum direct bilirubin, SI = serum iron, GLO = globulin, IBIL = serum indirect bilirubin, IgA = immunoglobulin A, IgM = immunoglobulin M, LDL-C = low-density lipoprotein cholesterol, TB = total bilirubin, TC = total cholesterol, UA = uric acid.
