# Supplementary material for: Predicting severe COVID-19 in elderly patients using routine laboratory indicators: Diagnostic accuracy of machine learning models
Source: Medicine (Baltimore). 2026 Jul 17;105(29):e49829. doi: 10.1097/MD.0000000000049829 (PMC13384693; doi:10.1097/MD.0000000000049829)
Supplement: Supplementary file 2 [file medi-105-e49829-s002.docx]

### ****Supplementary**** Table 2 Univariate Regression Analysis of the other 40 ****variables****

| **Risk Factor** | **B** | **S.E.** | **Wald** | **P** | **OR (95%CI)** |
| --- | --- | --- | --- | --- | --- |
| Chronic heart failure | 0.878 | 0.668 | 1.725 | 0.189 | 2.406 (0.649-8.920) |
|  | 0 |  |  |  | 1 |
| Chronic kidney disease | 0.703 | 0.549 | 1.638 | 0.201 | 2.020 (0.688-5.927) |
|  | 0 |  |  |  | 1 |
| Cerebral infarction | 0.524 | 0.455 | 1.330 | 0.249 | 1.689 (0.693-4.120) |
|  | 0 |  |  |  | 1 |
| Chronic liver disease | 0.932 | 1.244 | 0.561 | 0.454 | 2.541 (0.222-29.114) |
|  | 0 |  |  |  | 1 |
| Chronic respiratory disease | 0.33 | 0.447 | 0.546 | 0.460 | 1.391 (0.579-3.341) |
|  | 0 |  |  |  | 1 |
| Immune-mediated disease | -0.333 | 0.764 | 0.190 | 0.663 | 0.717 (0.160-3.206) |
|  | 0 |  |  |  | 1 |
| Gender (male) | -0.214 | 0.500 | 0.183 | 0.669 | 0.808 (0.303-2.152) |
|  | 0 |  |  |  | 1 |
| Diabetes mellitus | -0.182 | 0.452 | 0.163 | 0.687 | 0.833 (0.344-2.021) |
|  | 0 |  |  |  | 1 |
| Cardiovascular disease | -0.105 | 0.506 | 0.043 | 0.835 | 0.900 (0.334-2.424) |
|  | 0 |  |  |  | 1 |
| Hypertension | 0.026 | 0.438 | 0.004 | 0.952 | 1.027 (0.435-2.422) |
|  | 0 |  |  |  | 1 |
| Cancer (any) | -0.020 | 0.533 | 0.001 | 0.971 | 0.981 (0.345-2.785) |
|  | 0 |  |  |  | 1 |
| DBIL | 0.219 | 0.114 | 3.716 | 0.054 | 1.245 (0.996-1.555) |
| AST | 0.014 | 0.008 | 3.585 | 0.058 | 1.014 (0.999-1.030) |
| ALT | 0.020 | 0.011 | 3.535 | 0.060 | 1.020 (0.999-1.042) |
| GLO | -0.070 | 0.04 | 3.004 | 0.083 | 0.933 (0.862-1.009) |
| MO% | -0.094 | 0.057 | 2.713 | 0.100 | 0.910 (0.814-1.018) |
| EO% | -0.262 | 0.159 | 2.711 | 0.100 | 0.770 (0.564-1.051) |
| LDL-C | -0.371 | 0.227 | 2.671 | 0.102 | 0.690 (0.443-1.077) |
| SI | -0.053 | 0.036 | 2.160 | 0.142 | 0.948 (0.884-1.018) |
| UA | -0.003 | 0.002 | 2.073 | 0.150 | 0.997 (0.994-1.001) |
| BA% | -0.978 | 0.738 | 1.757 | 0.185 | 0.376 (0.089-1.597) |
| TB | 0.048 | 0.037 | 1.679 | 0.195 | 1.049 (0.976-1.128) |
| LY | -0.54 | 0.421 | 1.646 | 0.199 | 0.583 (0.255-1.330) |
| IgM | 0.452 | 0.357 | 1.602 | 0.206 | 1.571 (0.781-3.160) |
| MPV | 0.213 | 0.187 | 1.286 | 0.257 | 1.237 (0.857-1.786) |
| Age | 0.027 | 0.025 | 1.201 | 0.273 | 1.027 (0.979-1.078) |
| BA | 5.637 | 5.583 | 1.019 | 0.313 | 280.589 (0.005-15862164.249) |
| C4 | -1.776 | 1.805 | 0.968 | 0.325 | 0.169 (0.005-5.825) |
| EO | -1.400 | 1.646 | 0.724 | 0.395 | 0.247 (0.010-6.210) |
| IBIL | 0.041 | 0.054 | 0.570 | 0.450 | 1.042 (0.937-1.158) |
| MO | 0.498 | 0.665 | 0.560 | 0.454 | 1.645 (0.447-6.055) |
| IgA | -0.094 | 0.146 | 0.415 | 0.519 | 0.910 (0.683-1.212) |
| PLT | -0.001 | 0.002 | 0.206 | 0.650 | 0.999 (0.995-1.003) |
| MCHC | -0.008 | 0.019 | 0.167 | 0.683 | 0.992 (0.957-1.030) |
| PCT | -0.684 | 2.123 | 0.104 | 0.747 | 0.505 (0.008-32.382) |
| P-LCR | -0.006 | 0.023 | 0.072 | 0.789 | 0.994 (0.950-1.039) |
| MCV | 0.009 | 0.034 | 0.070 | 0.791 | 1.009 (0.945-1.078) |
| PDW | -0.008 | 0.066 | 0.013 | 0.909 | 0.992 (0.871-1.131) |
| Crea | 0 | 0.002 | 0.004 | 0.951 | 1.000 (0.996-1.003) |
| MCH | 0.004 | 0.093 | 0.002 | 0.968 | 1.004 (0.836-1.205) |

Variables with P>0.05 excluded from univariate regression analysis. DBIL = serum direct bilirubin, ALT = alanine aminotransferase, AST = aspartate aminotransferase, GLO = globulin, MO% = monocyte percentage, EO% = eosinophil percentage, LDL-C = low-density lipoprotein cholesterol, SI = serum iron, UA = uric acid, BA% = basophil percentage, TB = total bilirubin, LY = absolute lymphocyte count, IgM = immunoglobulin M, MPV = mean platelet volume, BA = absolute basophil count, C4 = complement C4, EO = absolute eosinophil count, IBIL = serum indirect bilirubin, MO = absolute monocyte count, IgA = immunoglobulin A, PLT = platelet count, MCHC = mean corpuscular hemoglobin concentration, PCT = plateletcrit, P-LCR = large platelet ratio, MCV = mean corpuscular volume, PDW = platelet distribution width, MCH = mean corpuscular hemoglobin.
